# Thrombosed external jugular vein aneurysm mimics to a branchial cyst: A novel case from Iraq and review of the literature

**DOI:** 10.1016/j.amsu.2021.102533

**Published:** 2021-06-29

**Authors:** Rawand A. Essa, Sirwan K. Ahmed, Dunya H. Bapir, Shero A. Rasul, Chawan P. Abubakr, Shiwan Q. Hamad

**Affiliations:** a Ph.D. in Cardiothoracic and Vascular Surgery, Lecturer in the University of Raparin, College of Nursing, Department of Adult Nursing, Rania, Sulaimani, Kurdistan-region, Iraq; b European Society for Thoracic Surgery (ESTS) Active Member, Iraq; c Department of Adult Nursing, College of Nursing, University of Raparin, Rania, Sulaimani, Kurdistan-region, Iraq; d Rania Teaching Hospital, Rania, Sulaimani, Kurdistan-region, Iraq; e Department of Medical Laboratory, College of Science, University of Raparin, Kurdistan-region, Iraq; f Rania Medical City Private Hospital, Rania, Sulaimani, Kurdistan-region, Iraq; g Department of Critical Care Nursing, College of Nursing, Urmia University of Medical Science, Iran; h Rania Pediatric & Maternity Teaching Hospital, Rania, Sulaimani, Kurdistan-region, Iraq

**Keywords:** External jugular vein aneurysm, Vein aneurysm, Thrombosis, Branchial cyst, Case report, Review of the literature, Surgical resection

## Abstract

**Introduction and importance:**

Venous aneurysms are rare diseases, and according to their locations, history will change. They will be diagnosed based on the clinical history and imaging modalities. The exact incidence of external jugular vein aneurysm remains controversial. In the neck, venous aneurysm has been reported most commonly in the internal jugular vein. Frequently the venous aneurysm has a fusiform shape, and the saccular type is extremely rare.

**Case presentation:**

Here we present a case of the external jugular vein aneurysm which was misdiagnosed as a branchial cyst presented with gradual swelling in the left supraclavicular region. The patient was diagnosed intraoperatively, and by histopathological examination. The patient successfully underwent surgery of Proximal and distal control of the external jugular vein without resection of the clavicle were performed, and *trans*-fixation of the external jugular vein was done without any complications.

**Clinical discussion:**

Idiopathic, spontaneous venous aneurysm of the external jugular vein thrombosis is extremely rare clinical findings. Computerized tomography was the gold standard test for the diagnosis of venous thrombosis, but nowadays neck ultrasound is the diagnostic test of choice. However, the mass was like a branchial cyst by ultrasound.

**Conclusion:**

External jugular vein aneurysm is rare, when it was a saccular type and thrombosed without any causes it will be extremely rare. When idiopathic thrombosis of external jugular vein aneurysm was confirmed by imaging modalities, then it was symptomatic, enlarged, ruptured or disfigured, the surgical excision will be mandatory without anticoagulant drugs preoperatively or postoperatively.

## Highlights

• More than 100 years ago venous aneurysm have been reported for the first time, and remains rare after one century.
• Computerized tomography was the gold standard test for the diagnosis of venous thrombosis, but nowadays neck ultrasound is the diagnostic test of choice. However, the mass was like a branchial cyst by ultrasound.
• When idiopathic thrombosis of external jugular vein aneurysm was confirmed by imaging modalities and investigations then it was symptomatic, enlarged, ruptured or disfigured, the surgical excision will be mandatory without any anticoagulant drugs preoperatively or postoperatively.
• In this paper a case of the external jugular vein aneurysm which was misdiagnosed as a branchial cyst with review of the literature is discussed.

## 1 Introduction

Venous aneurysms are rare diseases, and according to their locations, the history will change. They will be diagnosed based on the clinical history and imaging modalities [1,2]. In the neck venous aneurysms have been reported, most commonly in the internal jugular vein [1]. Frequently the venous aneurysm has a fusiform shape, the saccular type is extremely rare [1]. Venous aneurysms are usually symptomatic such as pain and swelling, but they rarely are asymptomatic like a painless mass [3]. Venous aneurysm is mostly caused by infection, trauma, venous obstruction (portal hypertension, thoracic outlet syndrome or neoplasm) [2], or catheterization, but spontaneous venous aneurysm is extremely rare [4]. Idiopathic external jugular vein thrombosis is extremely rare, it is usually due to infection, trauma, catheterization, or malignancy [4]. Nana et al., in his systematic review revealed that the primary indication of surgical treatment was aesthetic reasons and thrombosis was the most common EJVA complication. Most patients underwent more than one radiological examination [[5], [6], [7]]. The most prevalent therapy option is the open surgical procedure. We report a case of 28 years old female presented with idiopathic painless saccular thrombosed external jugular vein in the left side of the neck, which was misdiagnosed as a branchial cyst. The current study has been written in the line with SCARE 2020 criteria [8].

## 2 Case presentation

### 2.1 Patient information

On August 27, 2020, a 28-year-old woman, was admitted to the chest clinic in Rania Medical City (RMC) private hospital, she presented with gradual swelling in the left supraclavicular region, at the beginning the mass was painless, but within 1 month the pain started with no past medical and surgical history.

### 2.2 Clinical findings and diagnostic assessment

**Fig. 1:**
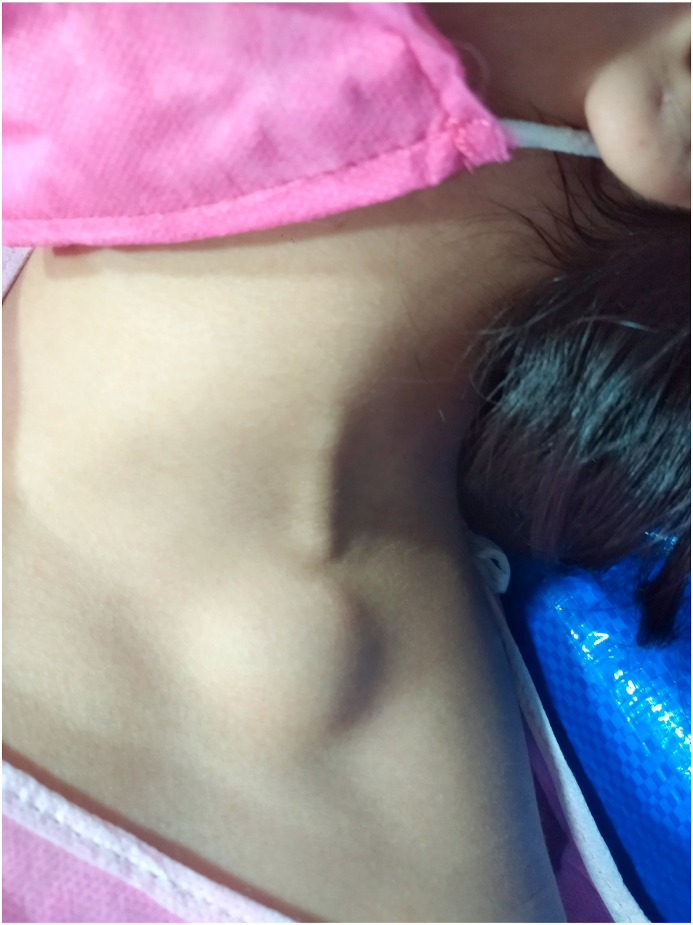
Painful, tender, non-pulsative mass at the left supraclavicular region.

Subsequently, physical examination revealed a hard, painful, tender, non-pulsative mass at the left supraclavicular region (Fig. 1), on the course of the external jugular vein, the mass in the first month was enlarging during lying on the bed. The skin overlying the mass did not have any signs of inflammation. The vital signs within normal limits. Regarding laboratory findings, there was no significant alterations. Additionally, there was no history of cervical trauma, cannulation of the neck vessels, prior hospital admission, or previous thromboembolic or oncological diseases. There was no cervical lymphadenopathy. The patient did not have symptoms in the otorhinolaryngological, thoracic or abdominal areas. She did not have night sweat, and neither weight loss, nor fever, no traveling recently. Chest, abdomen examination, and blood tests were normal. The patient was referred for a confirmatory US evaluation from a qualified radiologist, which revealed a 2 cm cystic mass without any increase in size with Valsalva maneuver, was not compressible. The mass was diagnosed as a branchial cyst in the neck.

### 2.3 Therapeutic intervention

On September 3, 2020, after explaining the procedure to patient the cardiothoracic and vascular surgeon with his team decided to surgery to remove a branchial cyst. On September 3, 2020, the patient underwent surgery by the first author, with local anesthesia in the supine position, with tilting of the neck toward right side and some extension. After good disinfection, and covering of the patient, through a transverse incision, the cyst was looks like venous aneurysm, black, uncompressible, and connected to the external jugular vein proximally. The cyst was extended toward the chest under clavicle. Due to an inflammatory reaction, there was adhesion around the cyst had been removed. Proximal and distal control of the external jugular vein without resection of the clavicle was performed. Ligate and *trans*-fixation of the external jugular vein was done without any complications. The cyst was excised, after opening the cyst intraoperatively it was clotted. Histopathological examination was made, and diagnosed as thrombosed external jugular vein aneurysm. On September 12, 2020, the stiches have been removed.

### 2.4 Follow up

After two days remained at critical care unit, and the patient was discharged to home for outpatient follow-up. Additionally, after 2 months, the patient did not have any additional local or general events during the follow-up period, and there was no recurrent swelling.

## 3 Discussion

The literature confirmed the rarity of this incidence. Venous aneurysms are either acquired or congenital [1]. Acquired venous aneurysms have many etiologies like tumors, iatrogenic, central venous catheterization, intravenous drug use [9], inflammation, infection, trauma, or idiopathic [10,11]. There are other causes like thrombophilia, Lumiere's syndrome [12], proximal humeral fracture, thoracic outlet syndrome, and aneurysms [[13], [14], [15]]. Thrombosis of the external jugular vein with unknown etiology is exceedingly rare, and the exact incidence is unknown [13,[16], [17], [18]]. Idiopathic thrombosed cases should undergo full examination, investigations and imaging till exclusion of all suspected causes will be done [19]. Therefore, we examined her, arranged well and sent her for full laboratory investigations and no thrombophilic disorders were found, and sent her for abdominal ultrasound, ultrasound of neck with Doppler; there were no underlying causes or malignancies apart from a cystic like lesion in the supraclavicular region. Thrombosis of the internal jugular vein was shown in the studies is more common than thrombosis of external jugular vein [4]. Thrombosis in lower limb veins is more common than other areas because of valves and effects of muscles around them while neck veins are more distensible and valveless so the thrombosis is rare [15]. Frequently the venous aneurysm has a fusiform shape, the saccular type is extremely rare [1]. Our case was a saccular type.

Clinical features of venous aneurysms are soft, non-tender or compressible swelling on the course of the vein [20]. Our patient had localized swelling in the left supraclavicular region on the course of external jugular vein, at the beginning the mass was painless but changed to painful and non-compressible mass after 3 months. Physical examination revealed a hard, painful, tender, non-pulsatile mass at the left supraclavicular region on the course of external jugular vein, the mass in the first month was enlarging during lying on bed and it was tender because of thrombosis. The differential diagnosis of a neck mass in an adult patient is composed of lymph node enlargement, carcinoma of neck organs, laryngocele, brachial cyst, dermoid cyst, arterial pseudoaneurysms, traumatic arteriovenous fistulas and rarely venous aneurysms [11,13,21,22]. Surgical treatment of a thrombosed vein is an effective choice because of the risk of pulmonary embolism or rupture [23,24]. Several authors confirmed that anticoagulation alone is insufficient for preventing such complications [[25], [26], [27], [28]]. Most patients with external jugular vein aneurysm underwent surgical treatment with local, and general anesthesia [16,[29], [30], [31], [32], [33], [34]].

Computerized tomography was the gold standard test for the diagnosis of venous thrombosis, but nowadays neck ultrasound is the diagnostic test of choice [1,14,35]. However, the mass was like a branchial cyst by ultrasound.

Complications of the neck veins are rare like thrombophlebitis, embolism or thrombosis, but still are threatening the life of patients [9] because of these complications, symptomatic patients, or cosmetic viewpoint a surgical excision of the lesion and ligation of the external jugular vein has been the standard treatment. Because the technique is less invasive, it can be performed under local anesthesia [35,36]. Moreover, in asymptomatic patient conservative management is preferable [2,33,[37], [38], [39]]. However, due to the scarcity of published research, there are no precise guidelines for the managing of these lesions.

## 4 Conclusion

External jugular vein aneurysm is rare, and if it was a saccular type, and thrombosed without any cause it will be extremely rare. When idiopathic thrombosis of the external jugular vein aneurysm was confirmed by imaging modalities and investigations then it was symptomatic, enlarged, ruptured or disfigured, surgical excision will be mandatory without any anticoagulant drugs preoperatively or postoperatively.

## Ethical approval

Ethical approval has been given by the ethics committee of our faculty.

## Funding

This research did not receive any specific grant from funding agencies in the public, commercial, or not-for-profit sectors.

## Author contribution

Rawand A. Essa: Conception and design, execution, analysis and interpretation of data, involved in drafting the article, revised it critically for important intellectual content, read and approved the final version of the manuscript. Sirwan K. Ahmed: Conception and design, execution, analysis and interpretation of data, involved in drafting the article, revised it critically for important intellectual content, read and approved the final version of the manuscript. Shero A. Rasul: involved in drafting the article, revised it critically for important intellectual content, read and approved the final version of the manuscript. Dunya H. Bapir: involved in drafting the article, revised it critically for important intellectual content, read and approved the final version of the manuscript. Chawan P. Abubakr: involved in drafting the article, revised it critically for important intellectual content, read and approved the final version of the manuscript. Shiwan Q. Hamad: involved in revised it critically for important intellectual content, read and approved the final version of the manuscript.

## Author agreement statement

We declare that this manuscript is original, has not been published before and is not currently being considered for publication elsewhere. We confirm that the manuscript has been read and approved by all named authors and that there are no other persons who satisfied the criteria for authorship but are not listed. We further confirm that the order of authors listed in the manuscript has been approved by all of us. We understand that the Corresponding Author is the sole contact for the Editorial process. He is responsible for communicating with the other authors about progress, submissions of revisions and final approval of proofs.

## Registration of research studies

Not applicable.

## Guarantors

Dr. Rawand A. Essa, and Registered Nurse Sirwan K. Ahmed: Accept full responsibility for the work and conduct of the study, had access to the data, and controlled the decision to publish.

## Consent

Written informed consent was obtained from the patient for publication of this case report and accompanying images. A copy of the written consent is available for review by the Editor-in-Chief of this journal on request.

## Provenance and peer review

Not commissioned, externally peer-reviewed.

## Declaration of competing interest

No competing interests were disclosed.

## References

[1] Drakonaki E.E., Symvoulakis E.K., Fachouridi A., Kounalakis D., Tsafantakis E. (2011). External jugular vein aneurysm presenting as a cervical mass. Int. J. Otolaryngol..

[2] Calligaro K.D., Ahmad S., Dandora R., Dougherty M.J., Savarese R.P., Doerr K.J., McAffee S., DeLaurentis D.A. (1995). Venous aneurysms: surgical indications and review of the literature. Surgery.

[3] Alshehri W.M., Alothmani S.K., Alshamrani A.M., ibrahim Almadhari R., Alqahtani A.S., Alrasheedi Saud D. (2019). Internal jugular vein aneurysm: a case report. J. Surg. Case Rep..

[4] Villanueva C.T., Ruiz J.R. (2019). Idiopathic external jugular vein thrombosis. Eur. J. Case Reports Intern. Med..

[5] Nana P., Gkrinia E., Maiou C., Karyda O., Korais C., Spanos K., Kouvelos G. (2021). Management of external jugular vein aneurysm: a systematic review. Vascular.

[6] ElKassaby M., Regal S., Khafagy T., El Alfy K. (2021). Surgical management of venous aneurysms. J. Vasc. Surg. Venous Lymphat. Disord..

[7] Gabrielli R., Rosati M.S., Siani A., Irace L. (2012). Management of symptomatic venous aneurysm. Sci. World J..

[8] Agha R.A., Franchi T., Sohrabi C., Mathew G., Kerwan A., Thoma A., Beamish A.J., Noureldin A., Rao A., Vasudevan B. (2020). The SCARE 2020 guideline: updating consensus Surgical CAse REport (SCARE) guidelines. Int. J. Surg..

[9] Battal B., Dursun E. (2008). External jugular vein aneurysm: clinical and radiologic imaging findings. Int. J. Head Neck Surg..

[10] Schatz I.J., Fine G. (1962). Venous aneurysms. N. Engl. J. Med..

[11] Hopsu E., Tarkkanen J., Vento S.I., Pitkäranta A. (2009). Acquired jugular vein aneurysm. Int. J. Otolaryngol..

[12] Karkos P.D., Asrani S., Karkos C.D., Leong S.C., Theochari E.G., Alexopoulou T.D., Assimakopoulos A.D. (2009). Lemierre's syndrome: a systematic review. Laryngoscope.

[13] Verma R.K., Kaushal D., Panda N.K. (2013). External jugular vein aneurysm with thrombus presenting as painful neck mass: a case report. Oman Med. J..

[14] Raju S., Byrne J. (2017). External jugular vein thrombosis secondary to deep tissue neck massage. J. Vasc. Surg. Cases Innov. Tech..

[15] Hindi Z., Fadel E. (2015). Idiopathic bilateral external jugular vein thrombosis. Am. J. Case Rep..

[16] Kim S.W., Chang J.W., Lee S. (2016). Unusual presentation of a cervical mass revealed as external jugular venous aneurysm. Vasc. Spec. Int..

[17] Pandey N.N., Sinha M., Deshpande A., Kumar S. (2020). External jugular vein aneurysm: successful endovascular management of an exceedingly rare entity. BMJ Case Rep..

[18] Pillai H.J., Roy N., Rao P.P., Shergill K.K., Shelly D., Badarudeen B. (2020). Isolated saccular aneurysm of the external jugular vein. Autops. Case Reports..

[19] Gbaguidi X., Janvresse A., Benichou J., Cailleux N., Levesque H., Marie I. (2011). Internal jugular vein thrombosis: outcome and risk factors. QJM An Int. J. Med..

[20] Verbeeck N., Hammer F., Goffette P., Mathurin P. (1997). Saccular aneurysm of the external jugular vein, an unusual cause of neck swelling. J. Belge Radiol..

[21] YiÄŸider A.P., KoÃ H.E., Ulusoy H.A., Keskin M. (2017). How to approach internal jugular vein aneurysm; A rare cause of neck mass, biomed. J. Sci. Tech. Res..

[22] Symvoulakis E.K., Klinis S., Peteinarakis I., Kounalakis D., Antonakis N., Tsafantakis E., Lionis C. (2008). Diagnosing a popliteal venous aneurysm in a primary care setting: a case report. J. Med. Case Rep..

[23] V Ioannou C., Kostas T., Tsetis D., Georgakarakos E., Gionis M., Katsamouris A.N. (2010). External jugular vein aneurysm: a source of thrombotic complications. Int. Angiol..

[24] Karapolat S., Erkut B., Ünlü Y. (2005). Multiple aneurysms of the left external jugular vein. Turk. J. Med. Sci..

[25] Dahl J.R., Freed T.A., Burke M.F. (1976). Popliteal vein aneurysm with recurrent pulmonary thromboemboli. Jama.

[26] Leu A.J., Canova C.R., Hoffmann U., Enzler M., Cassina P.C. (1999). A soft popliteal mass and pulmonary embolism. Eur. J. Vasc. Endovasc. Surg..

[27] Greenwood L.H., Yrizarry J.M., Hallett J.W. (1982). Peripheral venous aneurysms with recurrent pulmonary embolism: report of a case and review of the literature. Cardiovasc. Intervent. Radiol..

[28] Donald I.P., Edwards R.C. (1982). Fatal outcome from popliteal venous aneurysm associated with pulmonary embolism. Br. J. Radiol..

[29] El Husseiny M., Benhaiem N., Vayssairat M., Allaire E. (2011). Masson's vegetant intravascular hemangioendothelioma in an external jugular vein aneurysm with recurrent thrombosis. J. Vasc. Surg..

[30] Aiyappan S.K., Ranga U., Veeraiyan S. (2013). Aneurysm of external jugular vein mimicking hemangioma of neck. Indian J. Surg..

[31] Rajadurai A., Aziz A.A., Daud N.A.M., Wahab A.F.A., Muda A.S. (2017). Embolisation of external jugular vein aneurysm: a case report. Malaysian J. Med. Sci. MJMS..

[32] Garrachón J.M.E., Castañeira I.A., Gaspar B.E., Treceño J.L.A. (2012). Thrombosed phlebectasia of the external jugular vein with neck pain. Acta Otorrinolaringol. (English Ed.

[33] Parashi H.S., Rawekar K.H., Joshi M.M., Namdev H.S., Jadhao M.R., Bhosle K.N. (2018). Saccular aneurysm of external jugular vein with partial thrombosis. Asian Cardiovasc. Thorac. Ann..

[34] Neto T., Balhau R., Coelho L., Pinto I., Correia-Sá I., Silva Á. (2016). Thrombosed aneurysm of the external jugular vein: a rare cause of cervical mass. J. Craniofac. Surg..

[35] Chandran A., Kumar D., Kumar R., Sharma N., Kumar R. (2020). External jugular vein aneurysm: a rare cause of fluctuating neck swelling. Intern. Emerg. Med..

[36] Nana P., Korais C., Mpouronikou A., Lachanas V., Spanos K., Kouvelos G. (2020). Management of an external jugular vein aneurysm in a young patient. J. Vasc. Surg. Venous Lymphat. Disord..

[37] Gillespie D.L., Villavicencio J.L., Gallagher C., Chang A., Hamelink J.K., Fiala L.A., O'Donnell S.D., Jackson M.R., Pikoulis E., Rich N.M. (1997). Presentation and management of venous aneurysms. J. Vasc. Surg..

[38] Ekim H., Özen S. (2002). Primary venous aneurysm of the external jugular vein, East. J. Med..

[39] Lee H.Y., Cho S.H., Ko T.Y., Kim H.S., Kim J.I., Dal Park S., Cho S.R., Chun B.K. (2014). Saccular aneurysm of the external jugular vein: a case report. Korean J. Thorac. Cardiovasc. Surg..

